# Oral frailty in older adults: a scoping review of risk factors, adverse outcomes, and interventions

**DOI:** 10.1186/s12877-026-07470-2

**Published:** 2026-04-16

**Authors:** Jianjiao Yu, Liang Shang, Xiaorong Yang, Huolan Zhu, Xin Zhao, Ni Wang, Yulian Zhang, Weina Cao

**Affiliations:** 1 Xi Yuan Second Ward, Shaanxi Provincial People’s Hospital, Xian, 710068 Shaanxi China; 2 Department of Otorhinolaryngology, Shaanxi Provincial People’s Hospital, Xian, 710068 Shaanxi China; 3 Shaanxi Provincial Clinical Research Center for Geriatric Medicine, Shaanxi Provincial People’s Hospital, Xian, 710068 Shaanxi China; 4 Director’s Office, Shaanxi Provincial People’s Hospital, Xian, 710068 Shaanxi China; 5 Department of Rehabilitation Medicine, Shaanxi Provincial People’s Hospital, Xian, 710068 Shaanxi China

**Keywords:** Oral frailty, Older adults, Risk factors, Adverse outcomes, Interventions, Scoping review

## Abstract

**Background:**

Oral frailty (OF) is a significant geriatric syndrome, characterized by cumulative declines in oral function, with broad health implications. This scoping review synthesizes the existing evidence on the risk factors, adverse outcomes, and interventions related to OF in older adults.

**Methods:**

Following the PRISMA-ScR framework, we systematically searched PubMed, Embase, CINAHL, Web of Science, PsycINFO, and the Cochrane Library from inception to January 9, 2025. Two independent researchers conducted literature screening and data extraction, focusing on the first author, title, publication year, country, population, study design, sample size, scales, risk factors, adverse outcomes, and interventions.

**Results:**

A total of 589 records were identified, of which 64 studies met the inclusion criteria: 47 cross-sectional studies, 15 longitudinal studies, and 2 experimental studies. The analysis revealed OF risk factors across six domains: sociodemographic, physical/disease-related, oral health-related, dietary/nutritional, psychosocial, and lifestyle factors, with oral health-related factors being the most frequently reported. Adverse outcomes associated with OF included multidimensional health declines such as physical frailty/sarcopenia, malnutrition, cognitive impairment, oral dysbiosis, increased healthcare burden, and aspiration pneumonia, underscoring its systemic impact. Limited intervention evidence highlighted two promising programs: the CAMCAM program and the Oral Frailty Measures program, underscoring further intervention research.

**Conclusions:**

OF is a multifactorial syndrome with diverse adverse outcomes through bidirectional pathways. Future research should prioritize longitudinal designs to establish causality and develop integrated, culturally adapted prevention and intervention strategies.

**Supplementary Information:**

The online version contains supplementary material available at 10.1186/s12877-026-07470-2.

## Introduction

The global population is aging at an unprecedented rate, with projections indicating that individuals aged ≥ 60 years will reach 2.1 billion by 2050 [1]. This demographic transition underscores the urgent need to address geriatric syndromes that impair healthy aging, particularly those preventable or amenable to intervention. Among these, oral frailty (OF) has emerged as a critical yet underrecognized condition, playing a pivotal role in the overall health of older adults [2, 3].

OF is defined as a progressive decline in oral functions, including mastication, swallowing, speech, and salivation, and is intricately linked to systemic health deterioration [2, 3]. It represents a complex, multidimensional syndrome beyond a natural consequence of aging, exacerbating other age-related conditions and contributing to functional decline. The global prevalence of OF is alarmingly high, affecting approximately 29.5% of older adults worldwide [4]. This prevalence is even more pronounced in high-risk subgroups, such as hospitalized older patients with chronic conditions, including type 2 diabetes and cardiovascular diseases, where the incidence of OF can exceed 30.4% [5]. The multifactorial nature of OF, encompassing biological, behavioral, and socioeconomic determinants, makes it a challenging condition to address within the existing frameworks of geriatric care. Risk factors for OF include advanced age, female gender, comorbidities, tooth loss, periodontitis, malnutrition, depression, and so on [6–8]. OF not only impairs oral function but also establishes bidirectional relationships with various systemic conditions, including physical frailty, sarcopenia, cognitive decline, and malnutrition [9–11]. For example, impaired mastication limits dietary diversity, exacerbating malnutrition and accelerating muscle loss [12], while chronic oral inflammation worsens systemic inflammation and metabolic dysregulation, which in turn impacts cognitive and cardiovascular health [13]. These associations highlight the far-reaching consequences of OF, underscoring the need for a holistic approach to its prevention and management.

Despite its profound implications, OF remains insufficiently addressed in both the literature and clinical practice. Current research is fragmented across disciplines such as dentistry, geriatrics, and nutrition, hindered by inconsistent operational definitions, heterogeneous assessment tools (e.g., OFI-6, OFI-8), and a predominance of cross-sectional designs that limit causal inference [14]. While existing systematic reviews have provided valuable insights, they often focus narrowly on isolated dimensions of OF. For instance, Dibello et al. [3] systematically mapped the determinants of OF, and other important reviews, such as the scoping review by Parisius et al. [15] and the meta-analysis by Zhou et al. [16], have further elaborated on the concept and risk factors of OF. Subsequent reviews have strengthened the evidence linking OF to unfavorable outcomes [17, 18]. Nevertheless, a comprehensive synthesis that concurrently integrates evidence on the multifactorial risks, broad spectrum of adverse outcomes, and potential interventions for OF is still lacking. This fragmentation impedes the development of integrated, evidence-based strategies for preventing and managing OF.

A scoping review is well-suited to address this gap. This methodology allows for the delineation of a broad research scope, summarizes findings from diverse study designs, and identifies overarching patterns and research gaps in developing fields. Adhering to the Scoping Review Guidelines issued by the Joanna Briggs Institute (JBI) [14], this study aims to provide a comprehensive and updated map of the current evidence on OF in older adults. Specifically, this review concurrently explores three core components: (1) multidimensional risk factors, (2) diverse adverse outcomes, and (3) existing intervention strategies for OF. By integrating these components within the “OF” paradigm, this review seeks to inform the development of holistic early detection, prevention, and intervention strategies aimed at mitigating OF in older adults.

## Methods

### Protocol registration

The research protocol for this study was registered on the Open Science Framework (OSF) on 21 September 2024 (10.17605/OSF.IO/H4CNZ). The study adhered to the scoping review methodology outlined by Arksey and O’Malley, and the results were reported following the PRISMA ScR (PRISMA extension for scoping reviews) checklist [19].

### Search strategy

A comprehensive literature search was conducted across the following databases: PubMed, Embase, CINAHL, Web of Science, PsycINFO, and the Cochrane Library. Based on an initial literature review and our clinical and research expertise, we developed a search strategy that combined a set of subject headings (MeSH terms) and free-text keywords: “oral frailty” and “older adults”. The search strategy was intentionally designed to capture literature that explicitly conceptualized and investigated “OF” as a syndromic entity, ensuring conceptual coherence for this scoping review. Specific search terms were tailored to each database (supplementary file 1). Additionally, reference lists from included studies and relevant review articles were manually screened to identify further potentially eligible studies. The search included records from the inception of each database through 9 January 2025.

### Eligibility criteria

Inclusion and exclusion criteria for the literature review were determined based on the PCC framework (participants, concept, context). Inclusion criteria: ①Participants: Adults aged ≥ 60 years; ②Concept: OF, including its risk factors, adverse outcomes and interventions; ③Context: Any setting, including community, hospital or long-term care facilities. Exclusion criteria: ①Non-original research (e.g., reviews, conference abstracts, guidelines, books, etc.); ②Duplicate publications; ③Publications in languages other than English.

### Study selection

The retrieved literature was imported into EndNote X9 software, and duplicate records were removed. Two independent reviewers (Yu and Zhu) initially screened the first 30 articles to ensure consistent application of the inclusion criteria. Following this, the reviewers conducted an independent, parallel screening of titles and abstracts, adhering to the inclusion and exclusion criteria. A second screening of full texts was then performed to finalize the selection of studies for inclusion. Any discrepancies between the two reviewers were resolved through discussion with a third reviewer (Shang).

### Quality assessment of included studies

The methodological quality assessment was conducted independently by two reviews, with any discrepancies resolved through consensus or by a third reviewer: cross-sectional studies were evaluated using the Agency for Healthcare Research and Quality (AHRQ) criteria, with total scores of 0–3, 4–7, and 8–11 classified as low, moderate, and high quality, respectively [20]; cohort studies using the Newcastle-Ottawa Scale (NOS), with scores of 0–3, 4–6, and 7–9 corresponding to low, moderate, and high quality [21]; cluster randomized controlled trials (RCT) using the revised Cochrane risk of bias tool for randomized trials (RoB 2.0) for cluster-randomized trials, leading to a judgment of ‘low risk’, ‘some concerns’, or ‘high risk’ [22]; and quasi-experimental studies using the JBI checklist for quasi-experimental studies (9 items), where items were rated as ‘yes’, ‘no’, ‘unclear’, or ‘not applicable’ to determine overall quality [23].

### Data extraction and synthesis

Two independent reviewers manually extracted data from the included studies and summarized it in tabular form using Excel. The data extracted for each study included: first author, title, publication year, country, population, study design, sample size, scales, risk factors, adverse outcomes, and interventions. If necessary, the study authors were contacted to clarify missing or incomplete information. The extracted data were then thematically synthesized, with the identified domains categorized into risk factors, adverse outcomes, and interventions.

## Results

### Literature screening results

**Fig. 1:**
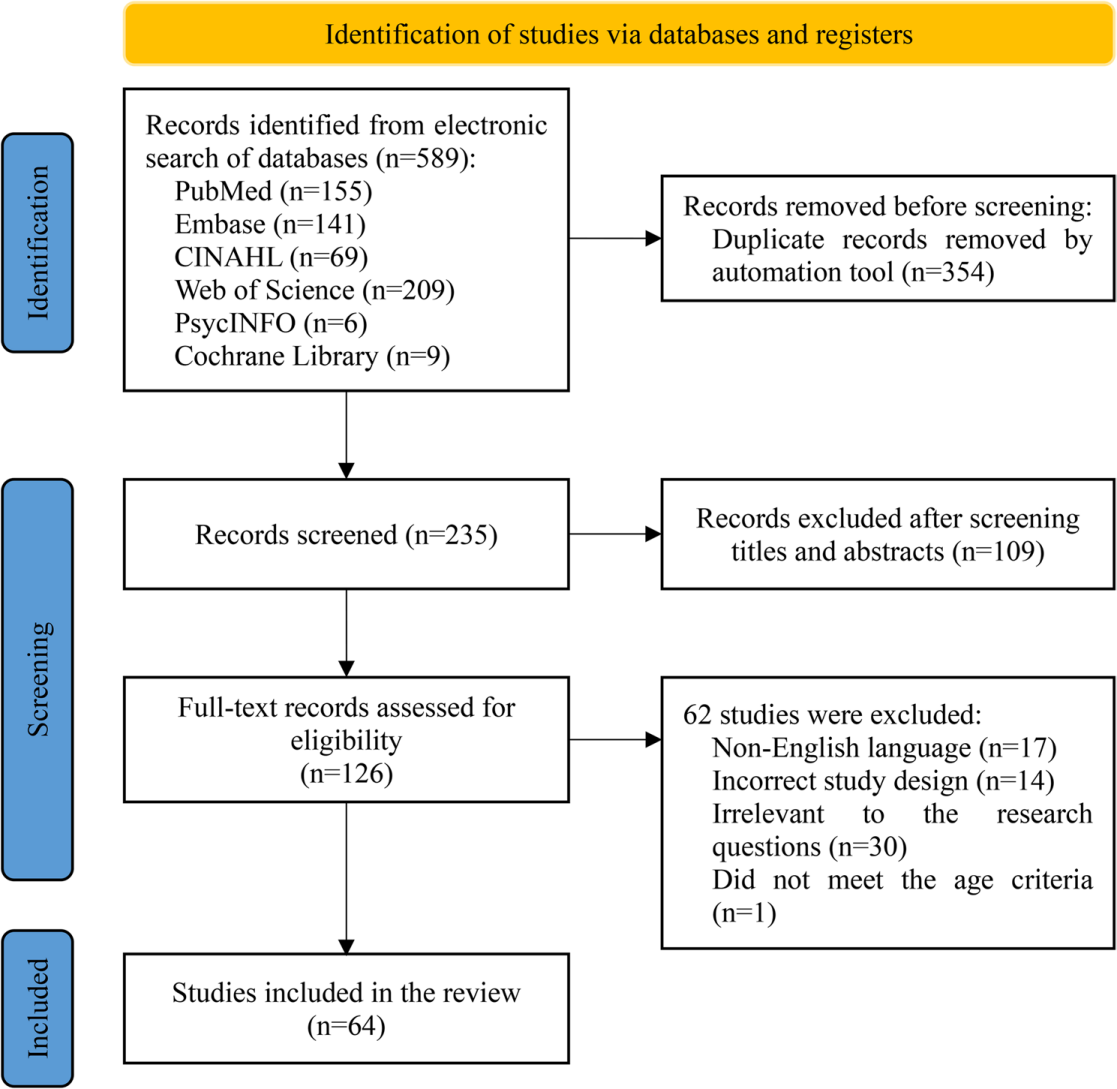
Literature screening flowchart

The initial search yielded a total of 589 articles. After removing 354 duplicate records, 235 articles were screened based on titles and abstracts. Of these, 126 articles were selected for full-text assessment. Ultimately, 64 studies met the inclusion criteria and were included in the final analysis. The flowchart for literature screening is shown in Fig. 1.

### Study characteristics

**Fig. 2:**
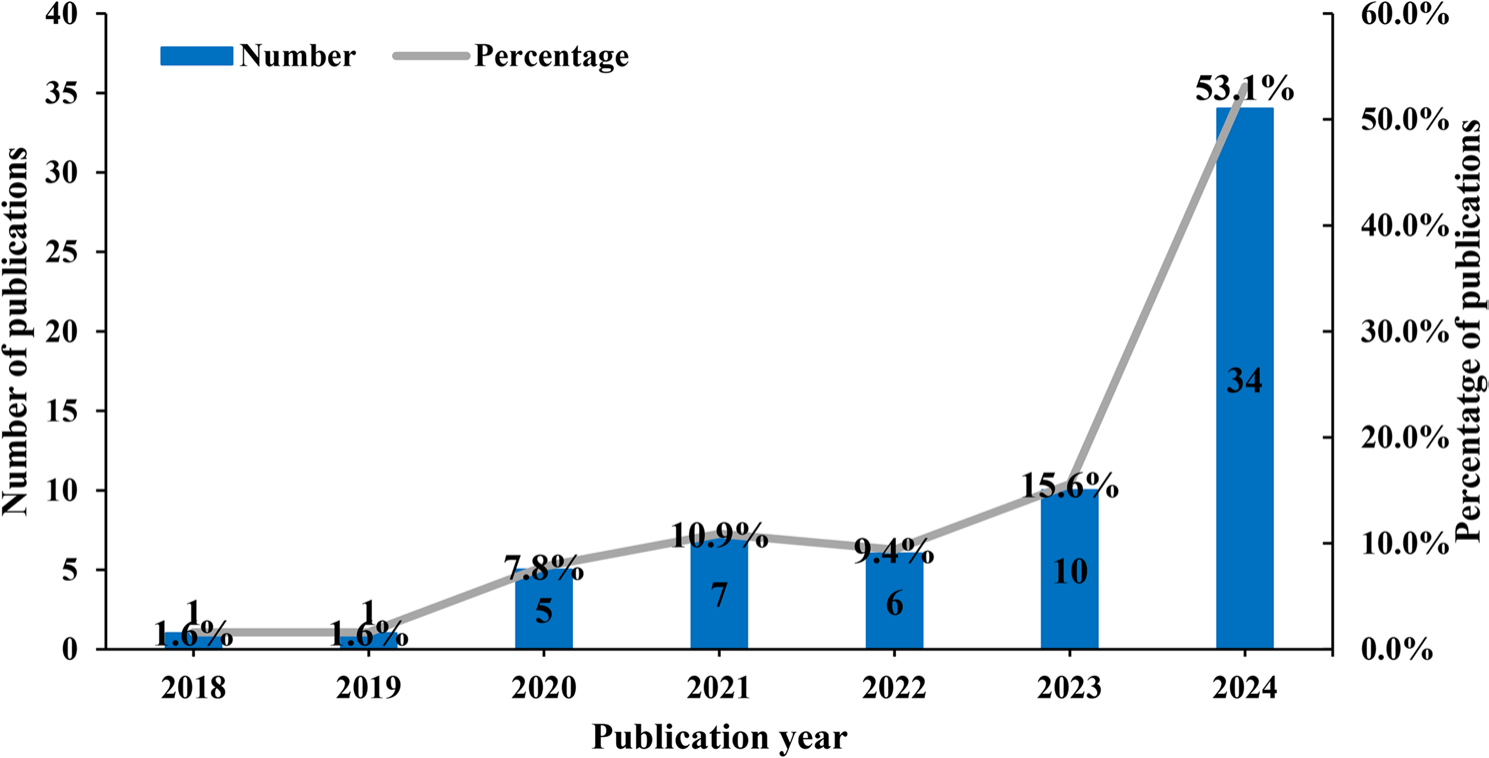
Annual number of publications on OF in older adults from 2018 to 2024

The included studies were published between 2018 and 2024, with 53.1% (34/64) published in 2024 (Fig. 2). The majority of studies were conducted in Japan (70.3%, 45/64), followed by China (21.9%, 14/64), Finland (6.3%, 4/64), and Korea (1.6%, 1/64). The Oral Frailty Index, specifically the OFI-8 and OFI-6 versions, was the most commonly used assessment tool (Table 1). The study designs comprised 47 cross-sectional studies (13 high quality, 34 medium quality by AHRQ criteria), 15 cohort studies (13 high quality, 2 medium quality by NOS), one cluster RCT (judged as high risk of bias by RoB 2.0), and one single-arm pre-post study (with a balanced quality rating per the JBI checklist) (supplementary file 2).

**Table 1:** Number of studies by OF instruments

Instruments	Number of studies
7- item OF questionnaire	2
OFI-6	21
3 items of KCL	4
OFI-8	25
OFr-5	1
A model to predict OF	1
OFr-6	2
2 subjective OF symptoms	1
OF-5	4
Functional tooth units	1
5 items of OF	1
7 items of OF	1

### Risk factors of OF

**Fig. 3:**
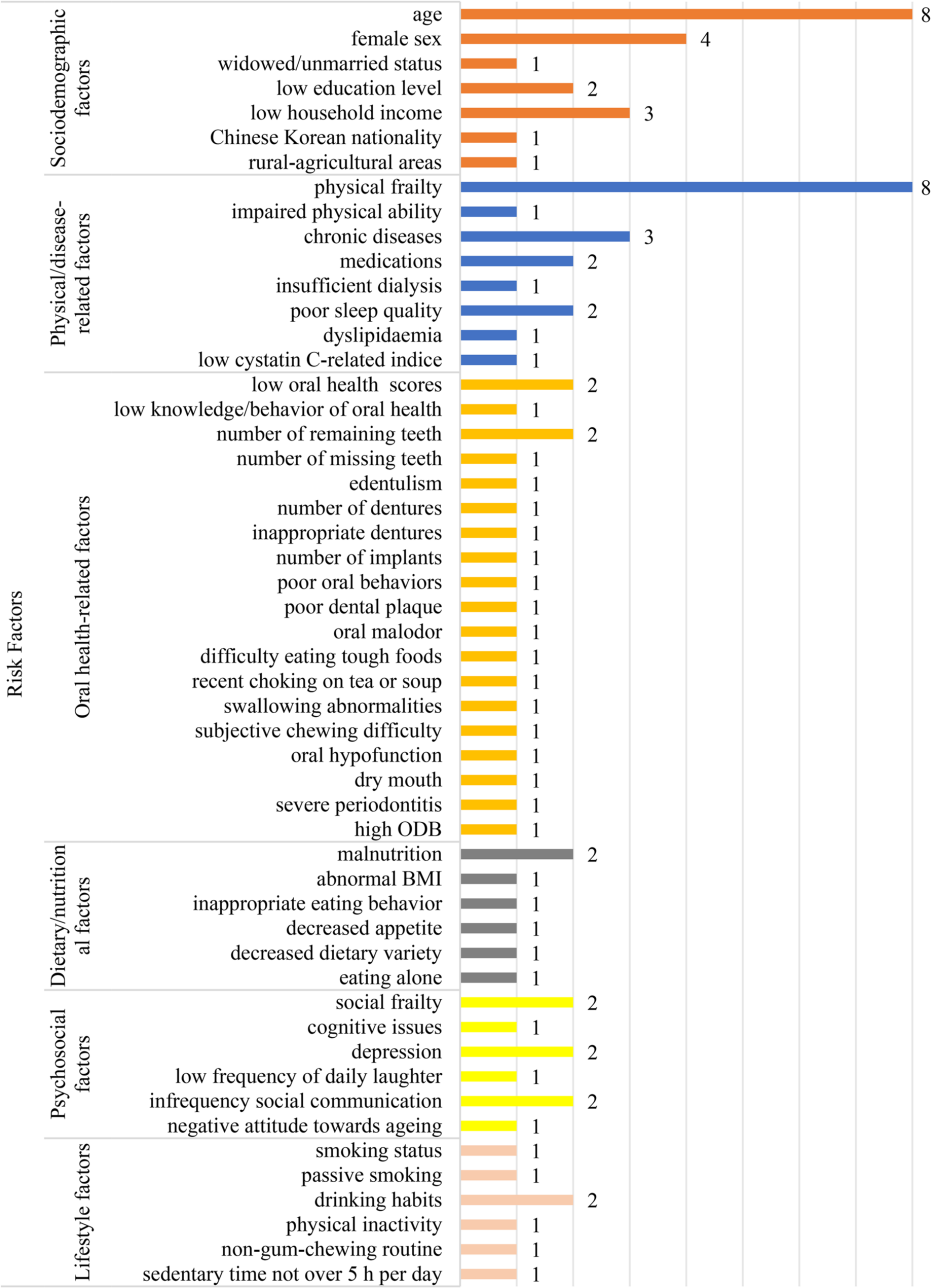
Frequency of risk factor reporting in included studies (*n* = 23)

Twenty-three studies explored the risk factors for OF in older adults, as detailed in Table 2. Figure 3 summarizes 46 distinct risk factors, which are categorized into six domains. The most frequently reported category is oral health-related factors.

**Table 2:** Characteristics of studies investigating risk factors for OF (*n* = 23)

Author, Year	Country	Population	Study design	Sample	Scale	Risk factors
Hihara et al. [29], 2019	Japan	Older patients of dental clinics	Cross-sectional study	744	7-item OF questionnaire	Age, inappropriate eating behavior
Ohara et al. [41], 2020	Japan	Community-dwelling older adults	Cross-sectional study	722	OFI-6	Eating alone Not dietary variety
Hasegawa et al. [28], 2020	Japan	Community-dwelling older adults	2-year prospective cohort study	427	3 items of KCL	Age, cognitive issues, impaired physical ability (slow walking speed and low knee extension)
Hironaka et al. [6], 2020	Japan	Community-dwelling older adults	Cross-sectional study	682	OFI-6	Age, social frailty, physical frailty, malnutrition, stroke, number of medications
Komatsu et al. [34], 2021	Japan	Community-dwelling older adults	Cross-sectional study	380	OFI-6	Physical frailty
Yamamoto et al. [25], 2022	Japan	Older patients of dental clinics	Cross-sectional study	843	OFI-6	Age, number of teeth present, difficulty eating tough foods compared with six months ago, recent history of choking on tea or soup
Lin et al. [7], 2022	China	Community-dwelling older adults	Cross-sectional study	1100	OFI-6	Depression
Izutsu et al. [36], 2023	Japan	Pre-frail community-dwelling older adults	Cross-sectional study	238	OFI-8	Diabetes mellitus, history of cancer, ill-fitting dentures, malnutrition
Kusunoki et al. [30], 2023	Japan	Patients of general internal medicine outpatient clinics	Cross-sectional study	374	OFI-8	Female sex, low cystatin C-related indices such as Cr/CysC and eGFRcys/eGFRcre levels
Nishimoto et al. [13], 2023	Japan	Disability-free, non-orally frail, community-dwelling older adults	6-year prospective cohort study	1234	OFI-6	Severe periodontitis
Chen et al. [32], 2024	China	Elderly maintenance hemodialysis patients	Cross-sectional study	325	OFI-8	Oral health score, Oral Health Knowledge score, Oral Health Behavior score, insufficient dialysis, social frailty, physical frailty, number of missing teeth, swallowing abnormalities Not Oral Health Belief score
Funakubo et al. [35], 2024	Japan	Community-dwelling older adults	Cross-sectional study	916	OFI-8	Low frequency of daily laughter, and social communication (daily talking and participation in community activities), depression, physical inactivity, physical frailty, smoking status, drinking habits
Ge et al. [24], 2024	China	Older patients with dental implants	Cross-sectional study	605	OFI-8	Age, female sex, low education level, low household income, number of implants, dyslipidemia
Hu et al. [26], 2024	China	Community-dwelling older adults	Cross-sectional study	380	OFI-8	Age, female sex, low education level, physical frailty, number of dentures, dry mouth, subjective chewing difficulties, oral health score, poor sleep quality
Julkunen et al. [38], 2024	Finland	Older adults in long-term care	Cross-sectional study	303	OFr-5	Edentulousness, high ODB
Kawamura et al. [42], 2024	Japan	Community-dwelling older adults	Cross-sectional study	1474	OFI-6	Non-gum-chewing routine
Maeda-Minami et al. [33], 2024	Japan	Community pharmacy visitors	Cross-sectional study	1386	OFI-8	Physical frailty, number of natural teeth (fewer than 20), benzodiazepine use
Morinaga et al. [39], 2024	Japan	Dental outpatients	Cross-sectional study	637	7-item OF questionnaire	Oral hypofunction
Nakagawa et al. [40], 2024	Japan	Late-stage older adults	Cross-sectional study	2727	OFI-6	Decreased appetite and dietary variety
Tamaki et al. [37], 2024	Japan	Community-dwelling 80-year-old adults	Cross-sectional study	3222	OFI-8	< 20 remaining teeth, poor oral behaviors [family dental clinic (no), and oral concerns (yes)], poor dental plaque, oral malodor
Wang et al. [8], 2024	China	Community-dwelling older adults	Cross-sectional study	478	OFI-8	Age, Chinese Korean nationality, female sex, low household income, number of chronic diseases, abnormal BMI, drinking, physical frailty, poor sleep quality, negative attitudes towards aging
Yamamoto et al. [31], 2024	Japan	Aged≥65y without long-term care requirements in the Japan Gerontological Evaluation Study	Cross-sectional study	165,164	A model to predict OF based on age, number of teeth, difficulty eating tough foods, and choking	Rural-agricultural areas, lower civic participation
Yin et al. [27], 2024	China	Community-dwelling older adults	Cross-sectional study	338	OFI-8	Passive smoking, widowed/unmarried status, physical frailty, aged 80 years and above, sedentary time Not smoking

#### Sociodemographic factors

Age [6, 8, 24–29], female sex [8, 24, 26, 30], widowed/unmarried status [27], low education level [24, 26], low household income [8, 24, 26], Chinese Korean nationality [8], and residence in rural-agricultural areas [31] were consistently identified as independent predictors of OF.

#### Physical/disease-related factors

Strong evidence linked physical frailty [6, 8, 26, 27, 32–35], impaired physical ability (e.g., slow walking speed and low knee extension) [28], number of chronic diseases [8], diabetes mellitus [36], history of cancer [36], stroke [6], number of medications [6], benzodiazepine use [33], insufficient dialysis [32], poor sleep quality [8, 26], dyslipidaemia [24], and low cystatin C-related indices such as Cr/CysC and eGFRcys/eGFRcre levels [30] to an elevated risk of OF.

#### Oral health-related factors

Lower Oral Health Assessment Tool (OHAT) scores [26, 32], reduced oral health knowledge and behavior scores [32], number of remaining teeth [33, 37], number of missing teeth [32], edentulism [38], number of dentures [26], ill-fitting dentures [36], number of implants [24], poor oral behaviors (e.g., no family dental clinic visits, oral concerns), poor dental plaque [37], oral malodor [37], difficulty eating tough foods [25], recent history of choking on tea or soup [25], swallowing abnormalities [32], subjective chewing difficulty [26], oral hypofunction [39], dry mouth [26], severe periodontitis [13] and high oral disease burden (ODB) [38] were all associated with increased risk of OF.

#### Dietary/nutritional factors

Key contributors to OF included malnutrition [6, 36], abnormal body mass index (BMI) [8], inappropriate eating behavior [29], decreased appetite [40], and decreased dietary variety [40]. Notably, one study found that eating alone was significantly associated with OF, rather than dietary variety [41].

#### Psychosocial factors

Significant psychosocial risks encompassed social frailty [6, 32], cognitive issues [28], depression [7, 35], low frequency of daily laughter [35], infrequency social communication (e.g., daily talking [35] and participation in community activities [31, 35]), and negative attitude towards ageing [8].

#### Lifestyle factors

Modifiable lifestyle risks included smoking status [35], passive smoking [27], and drinking habits [8, 35]. Interestingly, one study reported no significant association between smoking and OF, but passive smoking was identified as a risk factor with an odds ratio (OR) of around 2 [27]. Physical activity [35], gum-chewing routine [42], and sedentary time over 5 h per day are protective factors for OF [27].

### OF and adverse outcomes

Forty-one studies reported various adverse health outcomes associated with OF in older adults (Table 3). Below, we systematically categorize these outcomes by pathophysiological mechanism and evidence strength.

**Table 3:** Characteristics of studies reporting adverse outcomes associated with OF (*n* = 41)

Authors, Year	Country	Population	Study design	Sample	Scale	Adverse outcomes
Tanaka et al. [9], 2018	Japan	Community-dwelling older adults	4-year prospective cohort study	2011	OFI-6	Physical frailty, sarcopenia, disability, mortality
Hasegawa et al. [28], 2020	Japan	Older adults in Rural	2-year prospective cohort study	427	3 items of KCL	Social withdrawal
Iwasaki et al. [62], 2020	Japan	Community-dwelling older adults	Cross-sectional study	1054	OFI-6	Malnutrition
Nomura et al. [12], 2020	Japan	Community-dwelling older adults	Cross-sectional study	701	OFI-8	Insufficient nutritional intakes
Tanaka et al. [58], 2021	Japan	Community-dwelling older adults	8-year prospective cohort study	2011	OFI-8	Disability
Hiltunen et al. [43], 2021	Finland	Long-term care residents	Cross-sectional study	349	OFr-6	Physical frailty, dementia, malnutrition, lower BMI, chewing and swallowing difficulties, need for help with daily activities
Hoshino et al. [65], 2021	Japan	Community-dwelling older adults	Cross-sectional study	769	OFI-6	Reduced dietary variety
Iwasaki et al. [10], 2021	Japan	Community-dwelling older adults	2-year prospective cohort study	466	OFI-6	Deteriorating nutritional status
Iwasaki et al. [51], 2021	Japan	Community-dwelling older adults	Cross-sectional study	1082	OFI-6	Poor gait performance
Suzuki et al. [66], 2021	Japan	Community-dwelling older adults	Cross-sectional study	240	3 items of KCL	Decreased mineral intake
Baba et al. [70], 2022	Japan	Community-dwelling older adults	Cross-sectional study	210	OFI-6	Carriage of oral Candida
Ishii et al. [44], 2022	Japan	Diabetic patients	Cross-sectional study	111	OFI-8	Physical frailty
Kuo et al. [45], 2022	China	Rural middle-old community-dwelling people with cognitive decline	Cross-sectional study	308	OFI-8	Physical frailty
Lin et al. [7], 2022	China	Community-dwelling older adults	Cross-sectional study	1100	OFI-6	Depression
Doi et al. [59], 2023	Japan	Latter-stage older adult	6-year prospective cohort study	538	2 subjective OF symptoms	Disability, mortality, medical and dental expenditures
Nagatani et al. [67], 2023	Japan	Community-dwelling older adults	9-year prospective cohort study	244	OFI-6	New-onset mild cognitive impairment
Nakatani et al. [68], 2023	Japan	Older elective surgical patients	Cross-sectional study	331	OFI-8	Pre-operative undiagnosed cognitive impairment
Puranen et al. [61], 2023	Finland	Long-term care residents	3-year prospective cohort study	349	OFI-6	Health-related quality of life, survival Not energy and protein intake
Tanaka et al. [46], 2023	Japan	Community-dwelling older adults	9-year prospective cohort study	2031	OF-5	Physical frailty, disability, mortality
Teranishi et al. [64], 2023	Japan	Older patients after gastrectomy	3-month retrospective cohort study	60	Functional tooth units	Body weight loss
Arai et al. [73], 2024	Japan	Later-stage older adults	Cross-sectional study	2190	OFI-6	Medical and dental expenses
Chen et al. [75], 2024	China	Hospitalized older patients	Cross-sectional study	168	OFI-6	Aspiration pneumonia
Chen et al. [71], 2024	China	Hospitalized older patients	Cross-sectional study	103	OFI-6	Enterobacterales within the oral cavity
Fei et al. [11], 2024	China	Community-dwelling older adults	Cross-sectional study	307	OFI-8	Physical frailty, poor global cognitive function and executive function
Hu et al. [69], 2024	China	Older patients undergoing non-cardiac surgery	Cross-sectional study	303	OFI-8	Postoperative delirium
Ikuno et al. [76], 2024	Japan	Older patients undergoing abdominal visceral surgery	Retrospective cohort study	791	OFI-8	Postoperative infection
In-Ja et al. [74], 2024	Korea	Community-dwelling older adults	Cross-sectional study	1318	5 items of OF	Health-related quality of life
Iwasaki et al. [63],2024	Japan	Hemodialysis older patients	Cross-sectional study	152	OF-5	Malnutrition
Iwasaki et al. [47], 2024	Japan	Community-dwelling older adults	Cross-sectional study	1206	OF-5	Low dietary variety, social isolation, physical frailty
Kamide et al. [53], 2024	Japan	Community-dwelling older adults	Cross-sectional study	237	OFI-8	Fall risk
Kawamura et al. [50], 2024	Japan	Frailty clinic outpatients	Cross-sectional study	111	7 items of OF	Not sarcopenia
Kimura et al. [72], 2024	Japan	Community-dwelling older adults	Cross-sectional study	208	OFI-6	Prevotella percentage in the oral microbiota
Miyahara et al. [54], 2024	Japan	Older adults of research hospital frailty clinic	Cross-sectional study	248	OF-5	Malnutrition, fall risk
Miyasato et al. [49], 2024	Japan	Patients on hemodialysis	1-year prospective cohort study	201	OFI-8	Malnutrition, sarcopenia Not physical frailty
Puranen et al. [57], 2024	Finland	Long-term care residents	12-month prospective cohort study	254	OFr-6	Not fall risk
Song et al. [56], 2024	China	Community-dwelling older adults	Cross-sectional study	409	OFI-8	Fall risk, malnutrition
Watanabe et al. [60], 2024	Japan	Community-dwelling older adults	5.3-year prospective cohort study	11,374	OFI-8	Mortality
Xie et al. [52], 2024	China	Patients with cerebral small vessel disease	Cross-sectional study	126	OFI-8	Poor gait characteristics, falls
Yokoyama et al. [55], 2024	Japan	Community-dwelling older adults	2-year retrospective cohort study	7591	3 items of KCL	Falls
Yoneyama et al. [77], 2024	Japan	Community-dwelling older adults	Cross-sectional study	586	3 items of KCL	Lower urinary tract symptoms
Yu et al. [48], 2024	China	Older adults with T2DM	Cross-sectional study	292	OFI-8	HbA1c levels, nutritional status, physical frailty

#### Systemic physiological decline

Physical frailty emerged as the most frequently reported adverse outcome associated with OF in older adults [9, 11, 43–48]. Iwasaki et al. [47] proposed that low dietary variety and social isolation mediate the indirect association between OF and physical frailty. However, Miyasato et al. [49] found no such association in hemodialysis patients during a 1-year prospective cohort study. Concurrently, OF was identified as a risk factor for sarcopenia [9, 49], although Kawamura et al. [50] noted in a cross-sectional study that specific oral functions (oral diadochokinesis and tongue pressure), rather than OF itself, showed direct associations with sarcopenia.

OF was linked to poor gait performance [51, 52]. Participants with OF exhibited slower gait speed, shorter stride and step length, wider step width, and longer double support duration, along with higher variability in stride length and step length [51]. In patients with cerebral small vessel disease (CSVD), OF was associated with basic gait parameters (e.g., cadence, stride time, velocity, and length) during both single-task walking (STW) and dual-task walking (DTW) [52]. However, only the high-risk group showed significant variations in the coefficient of variation and phase coordination index during cognitive DTW, as compared to STW [52].

OF has been identified as a risk factor for fall incidents [52–56], independent of sarcopenia and physical performance [53]. Song et al. [56] demonstrated that OF was significantly associated with fall risk, with nutrition possibly acting as a mediating factor for the adverse effects of OF on fall risk. However, Puranen et al. [57] found no association between severe OF and falls among long-term care residents over a 12-month follow-up.

OF is also associated with a higher need for help with daily activities among older residents of long-term care facilities [43]. It showed strong predictive validity for disability [9, 46, 58, 59], mortality [9, 46, 59, 60], and reduced survival [61].

#### Malnutrition

OF significantly increases the risk of malnutrition [10, 43, 48, 49, 54, 56, 62, 63] and low BMI [43] in older adults, with particularly pronounced body weight loss observed post-gastrectomy [64]. It also reduces dietary variety [47, 65] and mineral intake, with the latter correlating with decreased bone mineral density [66]. While OF may generally contribute to insufficient nutritional intake [12], one study found no significant association between OF categories and energy/protein intake among long-term care residents [61].

#### Cognitive and mental impairment

OF could predict the risk of new-onset mild cognitive impairment [67], dementia [43], poor global cognitive function, and executive function [11], pre-operative undiagnosed cognitive impairment [68], and postoperative delirium [69]. Fei et al. [11] showed that the association between OF and poor global cognitive function and executive function was intensified by coexisting physical frailty. OF also promotes social withdrawal [28, 47] and dose-dependently increases the risk of late-life depression [7].

#### Oral health impairments

OF exacerbates oral dysbiosis, elevating the risk of Candida infections [70], Enterobacterales colonization (OR = 3.07; 5.58-fold higher with poor articulatory oral motor skill) [71], and Prevotella abundance [72]. It is also linearly correlated with chewing and swallowing difficulties among older residents in long-term care facilities [43].

#### Increased healthcare burden

OF increases medical and dental expenditures [59, 73] and reduces health-related quality of life, which declines linearly with the accumulation of OF signs [61, 74].

#### Other outcomes

Beyond primary outcomes, OF significantly predicts multisystem morbidity: Hospitalized older adults with swallowing tongue pressure < 10.32 kPa exhibit an elevated risk of aspiration pneumonia [75]; elective abdominal visceral surgery patients show a dose-responsive increase in postoperative infection risk with higher OFI-8 scores (OR = 1.10) [76]; OF independently increases the incidence of lower urinary tract symptoms (LUTS) (OR = 2.67) [77]; and in older adults with type 2 diabetes mellitus (T2DM), OF directly elevates HbA1c levels while indirectly affecting glycemic control through dual mediation pathways—physical frailty’s independent effect and a chain-mediated effect involving nutritional status and physical frailty [48].

### Prevention and interventions for OF in older adults

Current evidence on effective interventions for OF remains limited, with two primary programs documented (Table 4):

**Table 4:** Characteristics of intervention studies for OF (*n* = 2)

Authors, Year	Country	Population	Study design	Sample (intervention /control)	Scale	Duration	Intervention components
Shirobe et al. [78], 2022	Japan	Community-dwelling older adults with OF	A cluster RCT	51/32	OFI-6	12-week	Oral Frailty Measures Program: preparatory oral exercises, mouth-opening training, tongue pressure training, prosodic training, and masticatory training.
Hidaka et al. [79], 2023	Japan	Community-dwelling older adults	Single-arm pre-post comparison study	249	OFI-8	6-month	CAMCAM program: Participants gathered once a month at community centers to learn about nutrition, diet and oral health while eating a “munchy” textured lunch containing proper nutrition.

Shirobe et al. [78] found that the Oral Frailty Measures Program effectively alleviates OF in older adults with OF. This 12-week, cluster RCT involved community-dwelling older adults with existing OF. Participants in the intervention group (*n* = 51) received structured training sessions led by dentists and dental hygienists at baseline, 2, 4, and 8 weeks. Participants were also asked to continue the program voluntarily for an additional 12 weeks to assess its sustained effects. The program included preparatory oral exercises, mouth-opening training, tongue pressure training, prosodic training, and masticatory training. The study concluded that this program was effective in alleviating OF.

Hidaka et al. [79] developed the Comprehensive Awareness Modification of Mouth, Chewing, and Meal (CAMCAM) program. This 6-month, single-arm pre-post study was conducted at community centers (*n* = 249). Participants attended six monthly sessions (~ 30 min each), which combined education on oral health, oral function, nutrition, and food intake aimed at preventing OF. They also participated in eating a specially designed “CAMCAM textured lunch”, featuring harder food components (e.g., crushed almonds, raw cucumber/carrots, grain rice), cut into larger pieces with shortened cooking times, and providing balanced nutrition (27.5 g protein, 2.5 µg vitamin D, 2.2 g sodium, 595 kcal) that meets standards set for older adults. The findings of this pilot study suggest that the CAMCAM program may improve both oral and systemic frailty, as well as attitudes towards chewing, oral health, and meals, particularly in individuals with OF. The CAMCAM program shows promise as a community-based frailty prevention initiative and warrants further expansion.

## Discussion

This scoping review provides a contemporary and comprehensive synthesis of evidence on OF across 64 studies, further consolidating the understanding of OF as a multidimensional geriatric syndrome. While previous systematic reviews have made significant contributions to specific aspects of OF, such as its concept, prevalence, risk factors, and unfavorable outcomes [3, 15–18], our review distinguishes itself by its broad scope. It concurrently maps the multidimensional risk factors, the extensive spectrum of adverse outcomes, and the emerging evidence on interventions, thereby offering an integrated overview that underscores the interconnected nature of this syndrome.

A crucial point for interpreting this review’s findings is its specific scope. Our search strategy was designed to capture studies that explicitly used the term ‘OF’ or its direct synonyms. This approach ensured conceptual precision in mapping this emerging syndrome but necessarily excluded research on isolated oral dysfunctions (e.g., studies focusing solely on tooth loss, dysphagia, or xerostomia that did not frame them within the holistic OF paradigm). Therefore, the following synthesis and conclusions primarily reflect the associations and evidence gathered within this specific conceptual framework.

### Key determinants of OF: a multidimensional perspective

#### Sociodemographic factors

OF prevalence increases with age due to cumulative biological changes in oral structures and functions [6, 8, 24–29]. The observed female predominance in OF merits investigation, primarily attributed to estrogen deficiency [8, 24, 26, 30]. Postmenopausal estrogen decline accelerates bone calcium loss, increasing susceptibility to osteoporosis and alveolar bone atrophy. These changes reduce salivary secretion and flow rate while increasing vascular permeability, promoting conditions like halitosis, dental caries, and periodontal disease—key OF contributors. Furthermore, earlier permanent tooth eruption in females prolongs exposure to mechanical wear and cariogenic challenges. Gender-based disparities in healthcare-seeking behavior may compound this vulnerability. Being widowed or unmarried may increase the risk of OF, potentially due to reduced social support that can lead to diminished motivation for self-care and decreased access to dental care [27]. Socioeconomic disparities, including low educational attainment, low income, and rural residence, often mean limited access to preventive dental services, insufficient health literacy, and poorer overall health, all of which contribute to creating an environment conducive to the development of OF [8, 24, 26, 31].

#### Physical/disease-related factors

A robust bidirectional association exists between OF and physical frailty, with frail older adults being approximately three times more likely to develop OF than their non-frail counterparts [6, 8, 26, 27, 32–35]. Physical frailty can limit social engagement and reduce oral muscle activity, leading to decreased tongue pressure, impaired mastication and swallowing, and restricted tongue mobility, all of which contribute to OF. Moreover, impaired physical ability—such as slow walking speed and low knee extension—may hinder individuals from maintaining adequate oral hygiene or accessing dental care, further elevating OF risk [28]. Chronic diseases represent significant independent risk factors, with their cumulative number correlating with an elevated risk of OF [6, 8, 36]. For example, diabetes mellitus promotes caries through dry mouth and impaired swallowing function, whereas cancer therapies reduce salivary secretion, foster bacterial growth, and heighten susceptibility to periodontal disease and tooth loss [36]. Polypharmacy, common among individuals with chronic diseases, can also induce dry mouth and impair masticatory and swallowing functions [6, 8, 33]. Insufficient dialysis may cause systemic toxicity and metabolic imbalances—such as hypocalcemia—that accelerate frailty and compromise periodontal tissues, ultimately increasing OF risk [32]. Additionally, other systemic factors also contribute to OF risk. Poor sleep quality indirectly affects oral health by worsening physical frailty [8, 26]. Similarly, dyslipidemia disrupts blood circulation and immune function, reducing nutrient delivery and weakening oral tissue defenses against bacterial infections, thereby predisposing individuals to OF [24]. Reduced levels of cystatin C-related indicators, including Cr/CysC and eGFRcys/eGFRcre, are likewise strongly associated with an increased risk of OF, possibly due to their impact on muscle mass [30].

#### Oral health-related factors

Oral health determinants critically influence the development of OF. Lower OHAT scores strongly predict OF, reflecting reduced salivary secretion, diminished tongue pressure, and impaired masticatory and swallowing function—all accelerating OF progression [26, 32]. While inadequate oral health knowledge and behaviors significantly contribute to OF, oral health beliefs demonstrate no direct association [32]. Limited preventive awareness, cognitive impairment, and physical limitations reduce professional dental care utilization and promote harmful habits, thereby exacerbating dental caries, periodontal disease, and OF [32]. Diminished dentition, including fewer than 20 natural teeth [25, 33, 37], increased tooth loss [32], and complete edentulism [38], constitutes a major OF risk factor by directly impairing mastication, hindering nutrient absorption, and inducing perioral tissue atrophy. Prosthetic factors, such as the number of dentures [26], ill-fitting dentures [36], and the number of implants [24] further elevate OF risk. Dentures can accelerate alveolar bone resorption, compromise structural support, and create microgaps promoting fungal colonization; concurrent dry mouth and food impaction exacerbate OF [26]. Critically, well-fitting dentures maintain salivary flow and chewing efficiency, whereas ill-fitting dentures cause mucosal irritation, reduced salivation, and masticatory dysfunction—establishing a dose-dependent relationship between denture fit and OF severity [36]. Plaque accumulation and oral malodor represent additional OF risk factors, while tongue cleaning has been shown to improve respiratory and swallowing function in older adults [37]. Comorbidities including dry mouth [26] and severe periodontitis [13] significantly increase caries susceptibility, impair chewing/swallowing capacity, and drive tooth loss, ultimately compromising psychosocial well-being. Functional impairments, including difficulty eating tough foods [25], choking episodes [25], swallowing abnormalities [32], subjective chewing difficulty [26], oral hypofunction [39], and high ODB [38], trigger a degenerative cascade that directly disrupts swallowing mechanics and accelerates OF. Notably, family-supported dental care networks mitigate OF by enhancing health literacy, reducing treatment anxiety, and promoting preventive behaviors [37].

#### Dietary/nutritional factors

Dietary and nutritional factors play a critical role in the development of OF. Malnutrition is strongly associated with an increased likelihood of OF [6, 8, 36]. The vulnerability of type II muscle fibers—essential for swallowing—to nutritional deficiencies may explain this association [8]. While the relationship between OF and obesity remains inconclusive [8], obesity induces a pro-inflammatory state that can exacerbate the inflammatory response to plaque accumulation, thereby increasing the risk of periodontitis [80]. The eating process requires rapid and coordinated movements of the jaw, lips, and tongue from food intake to swallowing. Slow eating often reflects reduced masticatory function; thus, behaviors such as slow eating and poor chewing contribute to OF development [29]. Promoting appropriate eating habits, including effective chewing among older adults, is essential to prevent OF in a “super-aged” society. Reduced appetite and limited dietary diversity can create a vicious cycle: poor appetite restricts dietary variety, which further exacerbates OF [40]. Interestingly, one study found that eating alone, rather than reduced dietary diversity, was significantly associated with OF [41]. This may be attributed to decreased social interaction and reduced oral stimulation during meals. Future studies are needed to explore how OF contributes to this cycle and to develop interventions aimed at improving appetite, enhancing dietary diversity, and managing OF among older adults.

#### Psychosocial factors

Social frailty exerts a significant influence on OF in older adults [6, 32], where diminished self-esteem and poor treatment adherence reduce social interactions, consequently decreasing conversational opportunities and leading to reduced tongue pressure, weakened mastication, and slower tongue movement—all directly contributing to OF. Cognitive impairment [28] and depression [7, 35] have also been identified as independent risk factors for OF. Antidepressant use may exacerbate dry mouth and impair oral hygiene, increasing susceptibility to caries and periodontal disease, while depressive states often reduce toothbrushing frequency and hinder access to dental care. Infrequent daily laughter is associated with a higher risk of OF [35]. Laughter engages pectoral, abdominal, and facial muscles, enhances cognitive function through improved cerebral blood flow, and tones orofacial musculature—factors essential for OF prevention. Meanwhile, laughter alleviates depressive symptoms by reducing stress, thereby promoting better oral hygiene behaviors and lowering OF risk. Similarly, infrequent social communication, such as fewer daily talking [35], shorter conversation times, and low community activity participation [31, 35], further contributes to reduced tongue pressure and impaired oral function. Finally, attitudes toward aging play a critical role: negative perceptions discourage proactive oral health behaviors (e.g., regular dental visits), whereas positive outlooks support functional maintenance and help prevent OF [8].

#### Lifestyle factors

Modifiable lifestyle factors represent critical targets for the prevention and management of OF. Smoking is recognized as a risk factor for OF [35]. Interestingly, however, no significant association has been observed between active smoking and OF, whereas passive smoking has been identified as a risk factor, with an OR of approximately 2 [27]. Alcohol consumption disrupts the interaction between saliva and oral microbiota, leading to microbial imbalances and a reduction in beneficial lactic acid bacteria. Moreover, long-term heavy drinking increases the risk of bone loss, exacerbates periodontal disease, severely damages oral health, and accelerates OF progression [8, 35]. Physical inactivity is associated with a higher prevalence of OF [35], while self-reported sedentary behavior—particularly exceeding five hours daily—has shown a paradoxical protective effect [27]. This suggests that not all sedentary behaviors negatively impact health; some types may be beneficial. Future research should explore the relationship between specific sedentary behaviors and OF by distinguishing time spent in different activities. Notably, older adults who routinely chew gum exhibit a lower prevalence of OF [42]. One common reason for discontinuing gum chewing in later life is denture use; however, denture-friendly gum is now available. Incorporating a gum-chewing routine into daily life may help maintain oral function and mitigate OF risk.

### OF in older adults: an independent predictor of adverse outcomes?

The evidence synthesized strongly positions OF as an independent predictor and likely contributor to a cascade of adverse health outcomes in older adults.

#### Systemic physiological decline

Physical frailty is among the most common adverse outcomes associated with OF [9, 11, 43–48]. Iwasaki et al. [47] identified low dietary variety and social isolation as potential mechanisms linking OF to physical frailty. Conversely, Miyasato et al. [49] reported no significant association between OF and physical frailty among hemodialysis patients in a 1-year prospective cohort study. Older adults with OF often avoid hard-to-chew foods such as meats, fruits, and vegetables, which may further increase the risk of physical frailty. Additionally, OF has been implicated as a risk factor for sarcopenia [9, 49]. A cross-sectional study found no overall correlation between OF and sarcopenia; however, specific oral functions, such as oral diadochokinesis and tongue pressure, were associated with sarcopenia [50].

Gait impairment is another key manifestation of OF. Participants with OF demonstrate slower gait speed, shorter stride and step length, wider step width, longer double-support duration, and higher variability in stride and step length [51, 52]. Several mechanisms may explain this association. First, oral diseases can cause occlusal disturbances and jaw position changes, leading to alterations in head and neck posture and, ultimately, impairing postural control and balance. Second, malocclusion may reduce sensory input from the masticatory muscles and alveolar ligaments, compromising proprioception and postural stability. Third, occlusal imbalances can negatively affect muscle tone, which is closely linked to knee muscle performance and gait function. Fourth, abnormal oral habits, such as unilateral chewing dominance, may precede dental deterioration and promote dysfunctional muscle balance, disrupting overall body homeostasis. Finally, a nutritional pathway has been proposed, whereby inadequate dietary intake associated with OF leads to poor nutritional status, reduced muscle strength, and impaired gait performance.

OF also appears to increase fall risk [52–56], independent of sarcopenia and physical performance [53]. OF was significantly associated with fall risk, with nutrition potentially mediating this relationship [56]. However, no association was found between severe OF and falls during a 12-month follow-up [57]. OF may increase fall risk by influencing jaw position and, consequently, head and body posture and balance. Localized oral inflammation can trigger systemic inflammatory responses, accelerating muscle loss, sarcopenia, and fall risk. Additionally, older adults with oral weakness often limit intake of nutrient-rich foods such as meats, fruits, and vegetables, leading to malnutrition, sarcopenia, and subsequent falls [56].

Beyond mobility, OF has been linked to increased dependency in daily activities among older adults in long-term care facilities [43]. It strongly predicts disability [9, 46, 58, 59], mortality [9, 46, 59, 60], and reduced survival [61]. The underlying mechanism may involve oral bacterial infections (e.g., Streptococcus haematobium, Actinobacillus actinomycetemcomitans) triggering systemic inflammation, which exacerbates cardiovascular risk and contributes to higher mortality [60].

#### Malnutrition

Individuals with OF exhibit a higher prevalence and severity of malnutrition [10, 43, 48, 49, 54, 56, 62, 63]. This association is biologically plausible and consistent across various nutritional assessment methods. A major contributor to this relationship is the significant reduction in dietary variety among individuals with OF [47, 65]. Impaired chewing and swallowing, hallmark features of poor oral health, often lead to avoidance of nutrient-dense, hard-to-chew foods. Consequently, these individuals develop a preference for softer, carbohydrate-based foods while reducing protein-rich sources like meat, eggs, vegetables, and fruits. The reasons for decreased intake of soy and bean products remain unclear and warrant further investigation [65]. Avoidance of these essential food groups results in a less varied and unbalanced diet, characterized by lower intake of key nutrients, including protein (critical for muscle synthesis), vitamins, fiber, and minerals. Notably, reduced consumption of minerals such as potassium, magnesium, and phosphorus has been associated with decreased bone mineral density, even after adjusting for potential confounders [66]. This overall reduction in nutrient intake directly contributes to the risk of malnutrition. Although self-assessed oral function and tooth count showed minimal correlation with nutrient intake, positive oral health behaviors, such as brushing twice daily and maintaining regular dental visits, were significantly associated with better nutrient intake [12]. However, the lack of association between OF categories and energy/protein intake among long-term care residents may be attributed to methodological limitations, including insufficient analysis of the role of chewing and swallowing function in dietary choices within this context [61]. Additionally, low functional tooth units have been identified as a risk factor for postoperative weight loss in gastric cancer patients [64], emphasizing the clinical impact of impaired oral function on nutritional status.

#### Cognitive and mental impairment

OF is a significant predictor of cognitive decline in older adults, encompassing conditions such as new-onset mild cognitive impairment [67], dementia [43], poor global cognitive and executive function [11], pre-operative undiagnosed cognitive impairment [68], and postoperative delirium [69]. Longitudinal studies indicate that among individuals with physical frailty, OF markedly increases the risk of developing new-onset mild cognitive impairment, with contributing factors including tooth loss, chewing difficulty, and reduced tongue pressure [67]. The underlying mechanism operates through two primary pathways [67]: impaired masticatory function reduces cerebral blood flow and neural activity, particularly in the prefrontal cortex, which is strongly activated by chewing; simultaneously, feeding difficulties lead to decreased dietary diversity and subsequent malnutrition, indirectly promoting cognitive decline. OF predominantly affects executive function, an early marker of cognitive deterioration, and may therefore serve as a biomarker for the initial stages of executive dysfunction [11]. When OF coexists with physical frailty, the two conditions exert a synergistic effect, exacerbating both executive function and overall cognitive function [11]. The link between OF and preoperative cognitive impairment involves reduced activation of the prefrontal cortex—responsible for higher-order cognitive processes such as logical reasoning—due to impaired chewing movements, an effect particularly pronounced in older adults [68]. Chewing dysfunction associated with OF not only diminishes cerebral blood flow but may also compromise brain function indirectly via malnutrition and decreased neurotransmitter levels (e.g., acetylcholine), contributing to cognitive deficits. In the perioperative context, OF further promotes oral microbiota dysbiosis, which may elevate postoperative delirium risk through gut–brain axis mechanisms, underscoring the importance of preoperative oral health assessments in older patients [69]. Moreover, OF is associated with social withdrawal [28, 47] and exhibits a dose-response relationship with late-life depression [7], highlighting its multifaceted impact on health.

#### Oral health impairments

OF is closely associated with alterations in the oral microbiome [70–72]. Older adults with OF exhibit an increased risk of oral Candida colonization [70]. Additionally, the relative abundance of Prevotella in the oral microbiota is elevated among individuals with OF [72]. OF may also create conditions that favor colonization by Enterobacteriaceae in middle-aged and older adults [71]. Specifically, OF shows a 3.07-fold association with the presence of Enterobacterales in the oral cavity, and this risk increases to 5.58-fold in individuals with poor articulatory oral motor skills [71]. Enterobacteriaceae are native to the gastrointestinal tract; however, muscle weakness in the orofacial or oropharyngeal regions associated with OF, combined with swallowing difficulties, can lead to food or fluid retention in the pharynx, facilitating microbial exchange between the oropharyngeal and gastric flora [71]. Furthermore, OF demonstrates a linear relationship with chewing and swallowing difficulties among older residents of long-term care facilities [43].

#### Increased healthcare burden

OF contributes to reduced health-related quality of life [61, 74] and increased medical and dental expenditures [59, 73]. OF is associated with poor health, and visits to dental clinics for oral problems increase health care costs. Care should be provided, for example, through dental-medical multidisciplinary programmes, to minimize dental costs and prevent deterioration and exacerbation. OF may have caused not only the deterioration of general conditions such as malnutrition but also social frailty, which may have affected spiraling medical and dental expenditure [59].

#### Other outcomes

Older adults with OF are at heightened risk of aspiration pneumonia, particularly when tongue pressure falls below 10.32 kPa, a threshold identified as a reliable predictor [75]. This increased vulnerability stems from OF-related conditions such as dry mouth and poor oral hygiene, which promote pathogenic bacterial growth in the oral cavity; notably, evidence suggests that aspiration pneumonia can be effectively mitigated through enhanced oral hygiene and regular oral exercises [75]. OF is also a significant predictor of postoperative infections following elective abdominal visceral surgery [76], likely due to oral pathogenic bacteria, as many causative organisms for surgical site and intra-abdominal infections originate in the oral cavity, especially in patients with chronic inflammatory conditions such as periodontitis [81]. Although the direct relationship between OFI-8 scores and oral bacterial load remains unconfirmed, higher scores likely reflect increased bacterial colonization [76]. Furthermore, OF independently predicts LUTS through interconnected “nutrition-inflammation-frailty” pathways: impaired oral function reduces nutrient intake, accelerating physical frailty and sarcopenia, while chronic periodontitis induces systemic inflammation that disrupts bladder neuromuscular control [77]. Among older adults with T2DM, OF both directly and indirectly influences HbA1c levels via a chain-mediating effect involving nutritional status and physical frailty [48]. Poor oral health—particularly periodontitis—initiates local and systemic inflammation that exacerbates insulin resistance and elevates HbA1c, while dietary adaptations to oral dysfunction, such as a preference for soft foods with reduced fiber content, further impair glycemic control. Additionally, frailty-related chronic inflammation diminishes insulin sensitivity and alters basal metabolic rate, creating further challenges for glucose regulation.

### OF in older adults: treatment strategies

Evidence supporting specific interventions for OF remains limited, with only two structured programs identified in this review. The Oral Frailty Measures Program [78], a 12-week dentist-led regimen incorporating tongue pressure training, prosodic exercises, and masticatory rehabilitation, demonstrated significant symptom alleviation with sustained effects post-intervention. Similarly, the CAMCAM Program [79], which integrates nutritional education, functional training, and textured meals (e.g., harder foods providing 27.5 g of protein/595 kcal), yielded improvements in both oral and systemic frailty, as well as positive shifts in dietary attitudes. However, several limitations constrain the broader applicability of these interventions. Both programs fail to address psychosocial factors such as social isolation and depression, while the CAMCAM Program’s meal design lacks cultural adaptability, particularly in regions where traditional diets, such as the Asian soft-rice diet, predominate. Furthermore, the dentist-dependent models of these programs hinder scalability in resource-constrained settings.

Future management of OF requires integrated, multidimensional frameworks that reflect the syndrome’s complex nature. These frameworks must address the complex nature of the syndrome and include psychosocial support (such as communal dining to reduce the risk of eating alone), culturally adapted textured diets (retaining the protein/fiber principles of CAMCAM while substituting region-specific staples), and functional rehabilitation delivered via community health workers or digital platforms to enhance accessibility. Incorporating OF screening into surgical pathways is also critical to prevent aspiration pneumonia and postoperative infections. Moving forward, research must focus on rigorous randomized controlled trials comparing multidomain interventions with single-component approaches, incorporating cost-effectiveness analyses. Additionally, the development of standardized assessment tools for OF is necessary to address the current heterogeneity (e.g., OFI-6/OFI-8). Feasibility studies should also explore task-shifting strategies, such as training nurses to deliver basic oral exercises. Ultimately, bridging dentistry, nutrition, psychology, and geriatrics will be essential to transform OF from a biomarker of decline into a modifiable target for healthy aging.

### Implications for clinical practice and policy

The findings of this scoping review have several important implications for clinical practice and policy aimed at promoting healthy aging. First, there is an urgent need to integrate routine screening for OF into standard geriatric assessments conducted in community health centers, hospitals, and long-term care facilities. Given the strong association between OF and adverse outcomes like physical frailty, malnutrition, and cognitive decline, early identification using brief, validated tools is crucial for timely intervention. Second, our synthesis of risk factors provides a practical checklist for healthcare providers. Clinicians should be particularly alert to OF in older adults who are of advanced age, female, socially isolated, physically frail, or have poor oral hygiene, tooth loss, and dry mouth. Third, the identified interventions, though limited, offer a blueprint for action. The success of structured programs like the Oral Frailty Measures Program and the CAMCAM program underscores the value of combining oral functional training with nutritional guidance. These can be adapted and implemented by multidisciplinary teams involving dentists, dental hygienists, physicians, dietitians, and nurses. Finally, at a policy level, these findings argue for the development of integrated care models that bridge the silos between dental and medical healthcare systems. Public health initiatives should promote oral health literacy and community-based programs to prevent OF, ultimately reducing the broader healthcare burden associated with its systemic sequelae.

### Strengths and limitations

This scoping review provides a structured map of the evidence surrounding the specific concept of “OF” as a geriatric syndrome. Methodological rigor was ensured through adherence to PRISMA-ScR guidelines and preregistration of the protocol on OSF.

Nonetheless, several limitations should be noted. The most significant limitation stems from our search strategy. To maintain conceptual coherence for the syndromic entity of OF, we employed a focused set of terms centered on “oral frailty”. While this ensured precision, it undoubtedly resulted in the omission of a substantial body of relevant literature that investigates components of OF (e.g., mastication insufficiency, salivary hypofunction, dysphagia) without using the specific term. Consequently, our findings and conclusions are constrained to the literature that explicitly engages with the “OF” paradigm and may not fully represent the entire evidence base linking oral functional decline to health outcomes in older adults.

Second, the predominance of Japanese data limits generalizability. Third, heterogeneity in OF assessment tools complicates cross-study comparisons. Fourth, the heavy reliance on cross-sectional designs restricts causal inference. In addition, the scarcity of robust intervention trials and the exclusion of non-English literature may introduce bias.

Despite these limitations, this review offers essential insights into the current state of the science specifically addressing “OF” as a defined concept and informs future research and clinical practice within this framework.

## Conclusions

This scoping review highlights OF as a prevalent, multifactorial geriatric syndrome with profound implications for systemic health and functional independence. Driven by the interplay of sociodemographic, physical/disease-related, oral health, dietary/nutritional, psychosocial, and lifestyle factors, OF serves as both a marker of vulnerability and a modifiable risk factor for adverse outcomes such as frailty, sarcopenia, malnutrition, and cognitive decline. Despite its clinical significance, substantial gaps remain—particularly the lack of longitudinal data and evidence on effective, scalable interventions. Future research should focus on establishing causal relationships, developing standardized assessment tools, and designing integrated, culturally sensitive intervention strategies. Proactive OF screening and its incorporation into comprehensive geriatric care models are critical to mitigating adverse health outcomes and promoting healthy aging.

## Supplementary Information

Supplementary Material 1.

Supplementary Material 2.

## Data Availability

Data sharing is not applicable to this article, as no new data were generated for this study. The datasets used were derived from publicly available sources.

## Abbreviations

OF: Oral frailty
JBI: Joanna Briggs Institute
OSF: Open Science Framework
PRISMA ScR: PRISMA extension for scoping reviews
PCC: Participants, concept, context
AHRQ: Agency for Healthcare Research and Quality
NOS: Newcastle-Ottawa Scale
RCT: Randomized controlled trials
RoB: Risk of Bias tool
OHAT: Oral Health Assessment Tool
ODB: Oral disease burden
BMI: Body mass index
OR: Odds ratio
CSVD: Cerebral small vessel disease
STW: Single-task walking
DTW: Dual-task walking
LUTS: Lower urinary tract symptoms
T2DM: Type 2 diabetes mellitus
