# Supplementary material for: Oral frailty in older adults: a scoping review of risk factors, adverse outcomes, and interventions
Source: BMC Geriatr. 2026 Apr 16;26:747. doi: 10.1186/s12877-026-07470-2 (PMC13214296; doi:10.1186/s12877-026-07470-2)
Supplement: Supplementary file 1 — Supplementary Material 1. [file 12877_2026_7470_MOESM1_ESM.docx]

**Supplementary file 1**

**Search strategy for six databases**

| **1.PubMed: 155 Results** | | |
| --- | --- | --- |
| #1 | "aged"[MeSH Terms] OR"aged"[Title/Abstract] OR "elderly"[Title/Abstract] OR "senior*"[Title/Abstract] OR "geriatric*"[Title/Abstract] OR "older"[Title/Abstract] OR "elder*"[Title/Abstract] OR "old "[Title/Abstract] OR "aging "[Title/Abstract] | 5698706 |
| #2 | "oral frailty"[Title/Abstract] OR "oral frail*"[Title/Abstract] OR "oral weakness"[Title/Abstract] OR "oral vulnerability"[Title/Abstract] OR "oral debilit*"[Title/Abstract]) | 178 |
| #3 | #1 AND #2 | 155 |
| **2. Embase: 141 Results** | | |
| #1 | 'aged'/exp | 4147171 |
| #2 | 'elderly':ab,ti OR 'senior*':ab,ti OR 'geriatric*':ab,ti OR 'older':ab,ti OR 'elder*':ab,ti OR 'old':ab,ti OR 'aging':ab,ti | 3362225 |
| #3 | #1 OR#2 | 6488484 |
| #4 | 'oral frailty':ab,ti OR 'oral frail*':ab,ti OR 'oral weakness':ab,ti OR 'oral vulnerability':ab,ti OR 'oral debilit*':ab,ti | 162 |
| #5 | #3 AND #4 | 141 |
| **3. CINAHL: 69 Results** | | |
| S1 | MH (MH"aged") OR TI (aged OR elderly OR senior* OR geriatric* OR older OR elder* OR old OR aging) OR AB (aged OR elderly OR senior* OR geriatric* OR older OR elder* OR old OR aging) | 1359992 |
| S2 | TI ("oral frailty" OR "oral frail*" OR "oral weakness" OR "oral vulnerability" OR "oral debilit*") OR AB ("oral frailty" OR "oral frail*" OR "oral weakness" OR "oral vulnerability" OR "oral debilit*") | 73 |
| S3 | S1 AND S2 | 69 |
| **4. Web of Science: 209 Results** | | |
| #1 | TS= (aged OR elderly OR senior* OR geriatric* OR older OR elder* OR old OR aging) | 15049343 |
| #2 | TS= ("oral frailty" OR "oral frail*" OR "oral weakness" OR "oral vulnerability" OR "oral debilit*" ) | 243 |
| #3 | #1 AND #2 | 209 |
| **5.PsycINFO:6 Results** | | |
| S1 | TI(aged OR elderly OR senior* OR geriatric* OR older OR elder* OR old OR aging) OR AB(aged OR elderly OR senior* OR geriatric* OR older OR elder* OR old OR aging) | 691273 |
| S2 | TI("oral frailty" OR "oral frail*" OR "oral weakness" OR "oral vulnerability" OR "oral debilit*") OR AB ("oral frailty" OR "oral frail*" OR "oral weakness" OR "oral vulnerability" OR "oral debilit*") | 7 |
| S3 | S1 AND S2 | 6 |
| **6. Cochrane Library: 9 Results** | | |
| #1 | MeSH descriptor: [aged] explode all trees | 283161 |
| #2 | (elderly):ab,ti,kw OR (senior*):ab,ti,kw OR (geriatric*):ab,ti,kw OR (older):ab,ti,kw OR (elder*):ab,ti,kw OR (old):ab,ti,kw OR (aging):ab,ti,kw | 226903 |
| #3 | #1 OR #2 | 462894 |
| #4 | ("oral frailty"):ab,ti,kw OR (oral NEXT frail*):ab,ti,kw OR ("oral weakness"):ab,ti,kw OR ("oral vulnerability"):ab,ti,kw OR (oral NEXT debilit*):ab,ti,kw | 13 |
| #5 | #3 AND #4 | 9 |
