# Supplementary material for: Oral frailty in older adults: a scoping review of risk factors, adverse outcomes, and interventions
Source: BMC Geriatr. 2026 Apr 16;26:747. doi: 10.1186/s12877-026-07470-2 (PMC13214296; doi:10.1186/s12877-026-07470-2)
Supplement: Supplementary file 2 — Supplementary Material 2. [file 12877_2026_7470_MOESM2_ESM.docx]

**Supplementary file 2**

**Methodological quality assessment within studies**

| 1. ****Quality assessment of cross-sectional studies using AHRQ (n=47)**** | | | | | | | | | | | | | |
| --- | --- | --- | --- | --- | --- | --- | --- | --- | --- | --- | --- | --- | --- |
| ****Study**** | ****Q1**** | ****Q2**** | ****Q3**** | ****Q4**** | ****Q5**** | ****Q6**** | ****Q7**** | ****Q8**** | ****Q9**** | ****Q10**** | ****Q11**** | ****Score**** | ****Category**** |
| **Hihara et al^[29]^, 2019** | **Y** | **Y** | **N** | **Y** | **Y** | **N** | **Y** | **Y** | **U** | **N** | **U** | **6** | **Medium** |
| **Ohara et al^[41]^, 2020** | **Y** | **Y** | **Y** | **Y** | **Y** | **N** | **Y** | **Y** | **U** | **Y** | **Y** | **9** | **High** |
| **Hironaka et al^[6]^, 2020** | **Y** | **Y** | **Y** | **Y** | **Y** | **Y** | **Y** | **Y** | **U** | **Y** | **U** | **9** | **High** |
| **Komatsu et al^[34]^, 2021** | **Y** | **Y** | **Y** | **Y** | **Y** | **N** | **N** | **Y** | **U** | **Y** | **U** | **7** | **Medium** |
| **Yamamoto et al^[25]^, 2022** | **Y** | **Y** | **Y** | **Y** | **Y** | **N** | **N** | **Y** | **U** | **Y** | **U** | **7** | **Medium** |
| **Lin et al^[7]^, 2022** | **Y** | **Y** | **Y** | **Y** | **Y** | **Y** | **N** | **Y** | **U** | **Y** | **U** | **8** | **High** |
| **Izutsu et al^[36]^, 2023** | **Y** | **Y** | **Y** | **Y** | **Y** | **N** | **N** | **N** | **U** | **Y** | **U** | **6** | **Medium** |
| **Kusunoki et al^[30]^, 2023** | **Y** | **N** | **Y** | **Y** | **Y** | **N** | **N** | **N** | **U** | **N** | **U** | **4** | **Medium** |
| **Chen et al^[32]^, 2024** | **Y** | **Y** | **Y** | **Y** | **Y** | **N** | **N** | **N** | **U** | **Y** | **U** | **6** | **Medium** |
| **Funakubo et al^[35]^, 2024** | **Y** | **Y** | **Y** | **Y** | **Y** | **N** | **Y** | **Y** | **U** | **Y** | **U** | **8** | **High** |
| **Ge et al^[24]^, 2024** | **Y** | **Y** | **Y** | **Y** | **Y** | **Y** | **N** | **N** | **U** | **Y** | **U** | **7** | **Medium** |
| **Hu et al^[26]^, 2024** | **Y** | **Y** | **N** | **Y** | **Y** | **N** | **N** | **N** | **U** | **Y** | **U** | **5** | **Medium** |
| **Julkunen et al^[38]^, 2024** | **Y** | **Y** | **N** | **Y** | **Y** | **N** | **N** | **Y** | **U** | **Y** | **U** | **6** | **Medium** |
| **Kawamura et al^[42]^, 2024** | **Y** | **Y** | **Y** | **Y** | **Y** | **N** | **Y** | **Y** | **U** | **Y** | **U** | **8** | **High** |
| **Maeda-Minami et al^[33]^, 2024** | **Y** | **Y** | **Y** | **Y** | **Y** | **N** | **N** | **N** | **U** | **N** | **U** | **5** | **Medium** |
| **Morinaga et al^[39]^, 2024** | **Y** | **Y** | **Y** | **Y** | **Y** | **N** | **Y** | **N** | **U** | **Y** | **U** | **7** | **Medium** |
| **Nakagawa et al^[40]^, 2024** | **Y** | **N** | **Y** | **Y** | **Y** | **N** | **Y** | **Y** | **U** | **Y** | **U** | **7** | **Medium** |
| **Tamaki et al^[37]^, 2024** | **Y** | **Y** | **Y** | **Y** | **Y** | **N** | **N** | **Y** | **U** | **Y** | **U** | **7** | **Medium** |
| **Wang et al^[8]^, 2024** | **Y** | **Y** | **Y** | **Y** | **Y** | **N** | **N** | **N** | **U** | **Y** | **U** | **6** | **Medium** |
| **Yamamoto et al^[31]^, 2024** | **Y** | **N** | **Y** | **Y** | **Y** | **N** | **N** | **Y** | **Y** | **Y** | **U** | **7** | **Medium** |
| **Yin et al^[27]^, 2024** | **Y** | **Y** | **Y** | **Y** | **Y** | **N** | **N** | **Y** | **Y** | **Y** | **U** | **8** | **High** |
| **Iwasaki et al^[62]^, 2020** | **Y** | **Y** | **Y** | **Y** | **Y** | **N** | **Y** | **Y** | **U** | **Y** | **U** | **8** | **High** |
| **Nomura et al^[12]^, 2020** | **Y** | **N** | **Y** | **Y** | **Y** | **N** | **N** | **N** | **U** | **Y** | **U** | **5** | **Medium** |
| **Hiltunen et al^[43]^, 2021** | **Y** | **Y** | **Y** | **Y** | **Y** | **N** | **N** | **N** | **U** | **Y** | **U** | **6** | **Medium** |
| **Hoshino et al^[65]^, 2021** | **Y** | **Y** | **Y** | **Y** | **Y** | **N** | **Y** | **Y** | **U** | **Y** | **U** | **8** | **High** |
| **Iwasaki et al^[51]^, 2021** | **Y** | **Y** | **Y** | **Y** | **Y** | **N** | **N** | **Y** | **U** | **Y** | **U** | **7** | **Medium** |
| **Suzuki et al^[66]^, 2021** | **Y** | **Y** | **Y** | **Y** | **Y** | **N** | **N** | **Y** | **U** | **Y** | **U** | **7** | **Medium** |
| **Baba et al^[70]^, 2022** | **Y** | **N** | **Y** | **Y** | **Y** | **N** | **N** | **Y** | **U** | **Y** | **U** | **6** | **Medium** |
| **Ishii et al^[44]^, 2022** | **Y** | **Y** | **Y** | **Y** | **Y** | **N** | **N** | **N** | **U** | **Y** | **U** | **6** | **Medium** |
| **Kuo et al^[45]^, 2022** | **Y** | **Y** | **Y** | **Y** | **Y** | **N** | **Y** | **N** | **U** | **Y** | **U** | **7** | **Medium** |
| **Nakatani et al^[68]^, 2023** | **Y** | **Y** | **Y** | **Y** | **Y** | **N** | **N** | **Y** | **U** | **Y** | **U** | **7** | **Medium** |
| **Arai et al^[73]^, 2024** | **Y** | **Y** | **Y** | **Y** | **Y** | **N** | **N** | **Y** | **U** | **Y** | **U** | **7** | **Medium** |
| **Chen et al^[75]^, 2024** | **Y** | **Y** | **Y** | **Y** | **Y** | **N** | **N** | **N** | **U** | **N** | **U** | **5** | **Medium** |
| **Chen et al^[71]^, 2024** | **Y** | **Y** | **Y** | **Y** | **Y** | **Y** | **N** | **Y** | **U** | **Y** | **U** | **8** | **High** |
| **Fei et al^[11]^, 2024** | **Y** | **Y** | **Y** | **Y** | **Y** | **N** | **Y** | **Y** | **U** | **Y** | **U** | **8** | **High** |
| **Hu et al^[69]^, 2024** | **Y** | **Y** | **Y** | **Y** | **Y** | **N** | **N** | **N** | **Y** | **Y** | **U** | **7** | **Medium** |
| **In-Ja et al^[74]^, 2024** | **Y** | **Y** | **N** | **Y** | **Y** | **N** | **N** | **Y** | **U** | **N** | **U** | **5** | **Medium** |
| **Iwasaki et al^[63]^,2024** | **Y** | **Y** | **Y** | **Y** | **Y** | **N** | **Y** | **Y** | **U** | **Y** | **U** | **8** | **High** |
| **Iwasaki et al^[47]^, 2024** | **Y** | **Y** | **Y** | **Y** | **Y** | **N** | **Y** | **Y** | **U** | **Y** | **U** | **8** | **High** |
| **Kamide et al^[53]^, 2024** | **Y** | **Y** | **Y** | **Y** | **Y** | **N** | **N** | **Y** | **U** | **U** | **U** | **6** | **Medium** |
| **Kawamura et al^[50]^, 2024** | **Y** | **Y** | **Y** | **Y** | **Y** | **N** | **Y** | **N** | **U** | **Y** | **U** | **7** | **Medium** |
| **Kimura et al^[72]^, 2024** | **Y** | **Y** | **Y** | **Y** | **Y** | **N** | **N** | **Y** | **U** | **Y** | **U** | **7** | **Medium** |
| **Miyahara et al^[54]^, 2024** | **Y** | **Y** | **Y** | **Y** | **Y** | **N** | **N** | **N** | **U** | **Y** | **U** | **6** | **Medium** |
| **Song et al^[56]^, 2024** | **Y** | **Y** | **Y** | **Y** | **Y** | **N** | **N** | **Y** | **U** | **Y** | **U** | **7** | **Medium** |
| **Xie et al^[52]^, 2024** | **Y** | **Y** | **Y** | **Y** | **Y** | **N** | **N** | **N** | **U** | **Y** | **U** | **6** | **Medium** |
| **Yoneyama et al^[77]^, 2024** | **Y** | **Y** | **Y** | **Y** | **Y** | **N** | **Y** | **Y** | **U** | **Y** | **U** | **8** | **High** |
| **Yu et al^[48]^, 2024** | **Y** | **Y** | **Y** | **Y** | **Y** | **N** | **N** | **Y** | **U** | **Y** | **U** | **7** | **Medium** |
| **Note: Y = Yes (1point), N = No (0 point), U = Unclear (0 point).**  **Q1: Define the source of information (survey, record review)**  **Q2: List inclusion and exclusion criteria for exposed and unexposed subjects (cases and controls) or refer to previous publications**  **Q3: Indicate time period used for identifying patients**  **Q4: Indicate whether or not subjects were consecutive if not population-based**  **Q5: Indicate if evaluators of subjective components of study were masked to other aspects of the participants**  **Q6: Describe any assessments undertaken for quality assurance purposes (e.g.,test/retest of primary outcome measurements)**  **Q7: Explain any patient exclusions from analysis**  **Q8: Describe how confounding was assessed and/or controlled**  **Q9: If applicable, explain how missing data were handled in the analysis**  **Q10: Summarize patient response rates and completeness of data collection**  **Q11: Clarify what follow-up, if any, was expected and the percentage of patients for which incomplete data or follow-up was obtained** | | | | | | | | | | | | | |

| 1. ****Quality assessment of cohort studies using NOS (n=15)**** | | | | | | | | | | | |
| --- | --- | --- | --- | --- | --- | --- | --- | --- | --- | --- | --- |
| ****Study**** | ****Selection**** | | | | ****Comparability**** | ****Outcome**** | | | ****Score**** | ****Category**** |  |
|  | **Representativeness of the exposed**  **cohort** | **Selection of the nonexposed cohort** | **Ascertainment of exposure** | **Outcome was not present at start** | **Based on design**  **and analysis** | **Assessment**  **of outcome** | **Follow up**  **long enough** | **Adequacy of**  **follow up** |  |  |  |
| **Hasegawa et al^[28]^, 2020** | ***** | ***** | ***** | **-** | ****** | ***** | ***** | **-** | **7** | **High** |  |
| **Nishimoto et al^[13]^, 2023** | ***** | ***** | ***** | ***** | ****** | ***** | ***** | **-** | **8** | **High** |  |
| **Tanaka et al^[9]^, 2018** | ***** | ***** | ***** | ***** | ****** | ***** | ***** | ***** | **9** | **High** |  |
| **Tanaka et al^[58]^, 2021** | ***** | ***** | ***** | ***** | ****** | ***** | ***** | **-** | **8** | **High** |  |
| **Iwasaki et al^[10]^, 2021** | ***** | ***** | ***** | ***** | ****** | ***** | ***** | **-** | **8** | **High** |  |
| **Doi et al^[59]^, 2023** | **-** | ***** | ***** | ***** | ****** | ***** | ***** | **-** | **7** | **High** |  |
| **Nagatani et al^[67]^, 2023** | ***** | ***** | ***** | ***** | ****** | ***** | ***** | **-** | **8** | **High** |  |
| **Puranen et al^[61]^, 2023** | **-** | ***** | ***** | ***** | ****** | ***** | ***** | ***** | **8** | **High** |  |
| **Tanaka et al^[46]^, 2023** | ***** | ***** | ***** | ***** | ****** | ***** | ***** | ***** | **9** | **High** |  |
| **Teranishi et al^[64]^, 2023** | **-** | ***** | ***** | ***** | ***** | ***** | ***** | ***** | **7** | **High** |  |
| **Ikuno et al^[76]^, 2024** | **-** | ***** | ***** | ***** | ****** | ***** | ***** | ***** | **8** | **High** |  |
| **Miyasato et al^[49]^, 2024** | **-** | ***** | ***** | **-** | ****** | ***** | ***** | **-** | **6** | **Medium** |  |
| **Puranen et al^[57]^, 2024** | ***** | ***** | ***** | ***** | ****** | ***** | ***** | ***** | **9** | **High** |  |
| **Watanabe et al^[60]^, 2024** | ***** | ***** | ***** | ***** | ****** | ***** | ***** | ***** | **9** | **High** |  |
| **Yokoyama et al^[55]^, 2024** | **-** | ***** | ***** | ***** | ****** | **-** | ***** | **-** | **6** | **Medium** |  |

| 1. **Quality assessment of a cluster RCT study using RoB 2.0 tool for cluster-randomized trials**   **Shirobe et al^[78]^, 2022** | | | | | |
| --- | --- | --- | --- | --- | --- |
| **Bias domain and signalling question*** | **Response options** | | | | |
|  | **Y** | **PY** | **N** | **PN** | **NI** |
| **Randomization process** | | | | | |
| 1a.1 Was the allocation sequence random? | √ |  |  |  |  |
| 1a.2 Was the allocation sequence concealed until clusters were enrolled and assigned to interventions? | √ |  |  |  |  |
| 1a.3 Did baseline differences between intervention groups suggest a problem with the randomization process? |  |  |  | √ |  |
| Risk-of-bias judgment: low | | | | | |
| **Timing of identification or recruitment of participants** | | | | | |
| 1b.1 Were all the individual participants identified and recruited (if appropriate) before randomization of clusters? |  |  | √ |  |  |
| 1b.2 If N/PN/NI to 1b.1: Is it likely that selection of individual participants was affected by knowledge of the intervention assigned to the cluster? |  | √ |  |  |  |
| 1b.3 Were there baseline imbalances that suggest differential identification or recruitment of individual participants between intervention groups? |  |  |  | √ |  |
| Risk-of-bias judgment: high | | | | | |
| **Deviations from intended interventions** | | | | | |
| 2.1a Were participants aware that they were in a trial? | √ |  |  |  |  |
| 2.1b If Y/PY/NI to 2.1a: Were participants aware of their assigned intervention during the trial? |  | √ |  |  |  |
| 2.2 Were carers and people delivering the interventions aware of participants’ assigned intervention during the trial? | √ |  |  |  |  |
| 2.3 If Y/PY/NI to 2.1b or 2.2:Were there deviations from the intended intervention that arose because of the trial context? |  | √ |  |  |  |
| 2.4 If Y/PY to 2.3:Were these deviations likely to have affected the outcome? |  | √ |  |  |  |
| 2.5 If Y/PY/NI to 2.4:Were these deviations from intended intervention balanced between groups? |  |  |  | √ |  |
| 2.6 Was an appropriate analysis used to estimate the effect of assignment to intervention? |  |  | √ |  |  |
| 2.7 If N/PN/NI to 2.6:Was there potential for a substantial impact (on the result) of the failure to analyse participants in the group to which they were randomized? | √ |  |  |  |  |
| Risk-of-bias judgment: high | | | | | |
| **Missing outcome data** | | | | | |
| 3.1a Were data for this outcome available for all clusters that recruited participants? | √ |  |  |  |  |
| 3.1b Were data for this outcome available for all, or nearly all, participants within clusters? |  |  | √ |  |  |
| 3.2 If N/PN/NI to 3.1a or 3.1b: Is there evidence that the result was not biased by missing outcome data? |  |  | √ |  |  |
| 3.3 If N/PN to 3.2: Could missingness in the outcome depend on its true value? |  | √ |  |  |  |
| 3.4 If Y/PY/NI to 3.3: Is it likely that missingness in the outcome depended on its true value ? |  | √ |  |  |  |
| Risk-of-bias judgment: high | | | | | |
| **Measurement of the outcome** | | | | | |
| 4.1 Was the method of measuring the outcome inappropriate? |  |  | √ |  |  |
| 4.2 Could measurement or ascertainment of the outcome have differed between intervention groups? |  |  | √ |  |  |
| 4.3a If N/PN/NI to 4.1 and 4.2:Were outcome assessors aware that a trial was taking place? | √ |  |  |  |  |
| 4.3b If Y/PY/NI to 4.3a:Were outcome assessors aware of the intervention received by study participants? | √ |  |  |  |  |
| 4.4 If Y/PY/NI to 4.3b:Could assessment of the outcome have been influenced by knowledge of intervention received? |  | √ |  |  |  |
| 4.5 If Y/PY/NI to 4.4:Is it likely that assessment of the outcome was influenced by knowledge of intervention received? |  | √ |  |  |  |
| Risk-of-bias judgment: high | | | | | |
| **Selection of the reported result** | | | | | |
| 5.1 Were the data that produced this result analyzed in accordance with a pre-specified analysis plan that was finalized before unblinded outcome data were available for analysis? |  |  |  |  | √ |
| Is the numerical result being assessed likely to have been selected,on the basis of the results,from: |  |  |  |  |  |
| 5.2 ... multiple eligible outcome measurements (e.g.scales,definitions,time points) within the outcome domain? |  |  |  | √ |  |
| 5.3 ... multiple eligible analyses of the data? |  |  |  | √ |  |
| Risk-of-bias judgment: some concerns | | | | | |
| **Overall bias**: high | | | | | |
| Y=yes;PY=probably yes;PN=probably no;N=no;NA=not applicable;NI=no information. *Signalling questions for bias due to deviations from intended interventions relate to the effect of assignment to intervention. | | | | | |

| 1. **Quality assessment of a single-arm pre-post comparison study using JBI checklist for quasi-experimental studies**   Hidaka et al^[79]^, 2023 | | | | |  |
| --- | --- | --- | --- | --- | --- |
| **Evaluation item** | **Yes** | **No** | **Unclear** | **Not**  **applicable** | |
| 1.Is it clear in the study what is the “cause” and what is the “effect” (i.e. there is no confusion about which variable comes first)? | √ |  |  |  | |
| 2.Was there a control group? |  | √ |  |  | |
| 3.Were participants included in any comparisons similar? |  |  |  | √ | |
| 4.Were the participants included in any comparisons receiving similar treatment/care, other than the exposure or intervention of interest? |  |  | √ |  | |
| 5.Were there multiple measurements of the outcome, both pre and post the intervention/exposure? | √ |  |  |  | |
| 6.Were the outcomes of participants included in any comparisons measured in the same way? | √ |  |  |  | |
| 7.Were outcomes measured in a reliable way? | √ |  |  |  | |
| 8.Was follow-up complete and if not, were differences between groups in terms of their follow-up adequately described and analyzed? | √ |  |  |  | |
| 9.Was appropriate statistical analysis used? | √ |  |  |  | |
